# Anion-exchange membranes with internal microchannels for water control in CO_2_ electrolysis†

**DOI:** 10.1039/d2se00858k

**Published:** 2022-09-28

**Authors:** Kostadin V. Petrov, Justin C. Bui, Lorenz Baumgartner, Lien-Chun Weng, Sarah M. Dischinger, David M. Larson, Daniel J. Miller, Adam Z. Weber, David A. Vermaas

**Affiliations:** Department of Chemical Engineering, Delft University of Technology 2629 HZ Delft The Netherlands D.A.Vermaas@tudelft.nl; Department of Chemical Engineering, University of California Berkeley California 94720-1462 USA; Joint Center for Artificial Photosynthesis, Lawrence Berkeley National Laboratory California 94720-1462 USA azweber@lbl.gov

## Abstract

Electrochemical reduction of carbon dioxide (CO_2_R) poses substantial promise to convert abundant feedstocks (water and CO_2_) to value-added chemicals and fuels using solely renewable energy. However, recent membrane-electrode assembly (MEA) devices that have been demonstrated to achieve high rates of CO_2_R are limited by water management within the cell, due to both consumption of water by the CO_2_R reaction and electro-osmotic fluxes that transport water from the cathode to the anode. Additionally, crossover of potassium (K^+^) ions poses concern at high current densities where saturation and precipitation of the salt ions can degrade cell performance. Herein, a device architecture incorporating an anion-exchange membrane (AEM) with internal water channels to mitigate MEA dehydration is proposed and demonstrated. A macroscale, two-dimensional continuum model is used to assess water fluxes and local water content within the modified MEA, as well as to determine the optimal channel geometry and composition. The modified AEMs are then fabricated and tested experimentally, demonstrating that the internal channels can both reduce K^+^ cation crossover as well as improve AEM conductivity and therefore overall cell performance. This work demonstrates the promise of these materials, and *operando* water-management strategies in general, in handling some of the major hurdles in the development of MEA devices for CO_2_R.

## Abstract 2

We developed and simulated an anion exchange membrane with internal microchannels to hydrate the membrane. This allows water-consuming reactions (such as CO_2_ electrolysis) without sacrificing membrane conductivity or ion crossover.

## Graphical abstract

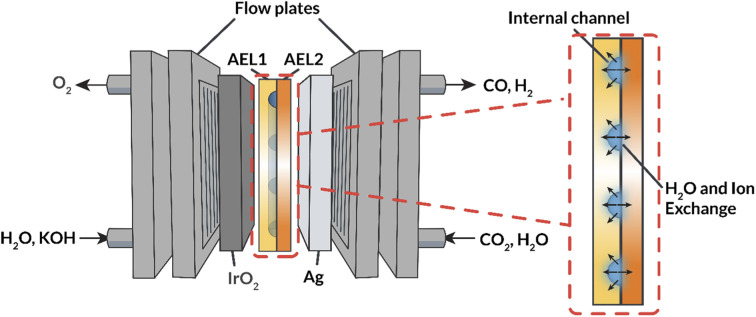

## Introduction

Large-scale carbon dioxide (CO_2_) electrolysis could be the key for storage of renewably-generated energy through sustainable production of fuels and chemicals.^1–3^ By applying an electric potential to an electrolyzer, CO_2_ and water can be electrochemically reduced at the cathode to form carbon monoxide (CO), formate, ethylene, methane, and other products, depending on the chosen catalyst.^4–9^ At the anode, oxygen is typically produced by oxidation of water.^10,11^ State-of-the-art scalable electrolyzers operate in a zero-gap configuration, where porous gas diffusion electrodes (GDE) are directly pressed against an ion-exchange membrane (also known as a membrane electrode assembly (MEA)),^2,12^ thereby minimizing transport losses. The GDE used on the cathode side facilitates direct supply of gaseous CO_2_ to the catalyst, which is advantageous because CO_2_ is sparingly soluble in water. If the reactor were to be operated in a fully aqueous medium, the CO_2_ reduction reaction (CO_2_RR) would quickly become mass-transport limited.^2,13^ Since a high current density is required for process scale-up, the membrane is a critical component since it must selectively conduct desired ions between the electrodes. The best reported results employ an anion-exchange membrane (AEM), which is shown to improve the faradaic efficiency (FE) of the process by enabling operation at alkaline pH.^14,15^

One of the challenges with this system is that the CO_2_RR consumes water in neutral or alkaline environments.^16^ This phenomenon can lead to the dehydration of the membrane surface close to the cathode, thereby inhibiting ionic transport and reducing overall efficiency. Additionally, the electro-osmotic flux of water away from the cathode when using an AEM, as well as the consumption of water by the CO_2_RR both can lead to reduced water availability for the electrochemical reactions within the cathodic catalyst. Therefore, water management is of utmost importance to ensure optimized conductivity of the membrane and ionomer materials, as well as to promote the desired CO_2_RR by negating the aforementioned challenges.^15,17^ Liu *et al.*^18^ demonstrated that several factors influence membrane water content, including reaction rate, water diffusion from the anode and the hydrated CO_2_ gas stream, and electro-osmotic flow. When the anolyte was deionized water, their cell lacked operational stability beyond 100 h. However, with 10 mM KHCO_3_ anolyte, their electrolyzer was able to operate at a stable voltage for 4000 h. This result reveals that water diffusion from the anode is not sufficient by itself to maintain membrane hydration at the cathode/membrane interface, but the contribution of the water molecules in the hydration shell of K^+^ ions, which cross over *via* electro-osmosis, ensured stable membrane hydration and water availability by the cathode surface. In a broader framework, the management of water transport in these devices has shown to be a strong lever for boosting the energy efficiency in alkaline water electrolyzers^19^ and increasing the stability of CO_2_ electrolyzers.^14,16,20–22^

Another phenomenon that can be detrimental to CO_2_RR is salt deposition on the cathode, which can block diffusion pathways and/or the catalyst surface.^15,23^ KHCO_3_, and K_2_CO_3_ salts deposition occurs at high K^+^ concentrations near the cathode, where CO_2_ also reacts with OH^−^ (produced in the CO_2_RR) to form (bi)carbonate.^17^ A decreased water content near the cathode surface will also promote salt deposition. To mitigate the crossover of K^+^, researchers have opted to limit the anolyte concentration to 10 mM.^18,24^ Such low cation concentrations in the anolyte minimize crossover, not only because they decrease the driving force for crossover (*i.e.*, electrochemical potential gradient), but also increase the membrane selectivity due to more effective Donnan exclusion.^25^ However, low anolyte concentrations may also result in poor ionic conductivity in the catalyst region.

**Fig. 1:**
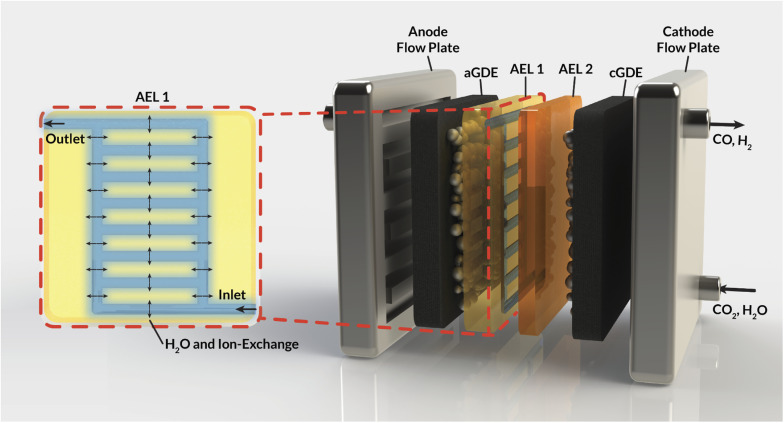
Schematic representation of an MEA cell for CO_2_RR that employs a bilayer AEM with internal channels to manage water fluxes. Inset (red box): cross-section depicting internal water channels in the AEM.

This work aims to address these challenges by introducing internal microchannels into the AEM (Fig. 1) that enable the circulation of water or electrolyte within the AEM. Using a mixture of continuum-scale modeling and experiments, our work demonstrates that these internal microchannels maintain AEM hydration by providing a direct supply of water to the cathode, enabling higher current densities for CO_2_RR. Furthermore, the impact of the flowing electrolyte inside these channels on the overall AEM conductivity and selectivity is assessed. Specifically, the impact of the channel geometry, position, and composition on the electrochemical performance of a CO_2_ electrolyzer is studied, expressed in terms of ohmic losses and observed ion crossover. Ultimately, this study sets the stage for the development of membrane materials with *operando* water-management strategies that will be critical in the deployment of CO_2_RR devices at scale.

## Methods

### Macroscale modeling

**Fig. 2:**
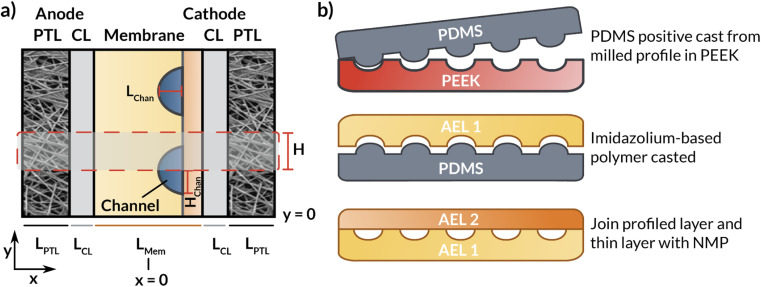
(a) Schematic representation of the simulated AEM-MEA in which the AEM has internal microchannels. (b) Schematic representation of the fabrication process and cross section of the entire bilayer AEM. The dashed box represents the modeled domain.

This section describes the modeling methods, governing equations, and assumptions used to model the electrochemical performance and local hydration of MEA devices employing AEMs with internal microchannels. Because hydration gradients are greatest for a full MEA, (*i.e.*, one with a vapor phase anodic feed), the AEM-MEA is modeled with a 100% relative-humidity (RH) water vapor feed in a nitrogen (N_2_) carrier gas. The domain modeled is a two-dimensional (2-D) representation of the full MEA device (Fig. 2a), including both anodic and cathodic porous-transport layers (PTLs), where the gaseous species diffuse through and distribute to the porous anodic and cathodic catalyst layers (which are modeled to be comprised of porous catalyst particles in an ionomer binder, see Fig. S1†) (CLs), where the electrochemical reactions occur. It is important to note that multiphase transport exists within the CL domains, where there is ionic transport within the ionomer in the CL, bulk vapor and liquid phase transport in the pore space of the CL, and electronic transport in the solid domain of the CL.^17^

In between the anodic and cathodic CLs is the AEM with internal microchannels, which is modeled with quarter-circular channels. The geometry of the channel is chosen to match the membrane fabricated by the process depicted in (Fig. 2b) and detailed in the following subsections. Because the embedded channels are symmetrically patterned, half of a repeating unit is selected to be simulated (red box in Fig. 2a). While this treatment does neglect edge effects, by applying symmetry conditions at the upper and lower boundaries of the modeled domain, the AEM can be more efficiently simulated in 2-D, greatly reducing computational cost.

The model captures the gradients in electrostatic potential and bulk ionic current through a secondary current distribution, the diffusive transport of concentrated gas species in the CLs and PTLs, bulk aqueous and vapor fluid flow through the porous media, and water transport throughout the CL and AEM domains. The governing equations describing these phenomena are discussed in detail in the following subsections.

### Charge transport

Charge transport within the ionomer, membrane, and electrode domains is modeled by use of a secondary current distribution, which accounts for the effect of hydration-dependent solution resistance as well as the effects of electrode kinetics. Secondary current distributions neglect concentration-dependent kinetics, such as the migration and diffusion of ionic species, and the consumption and generation of CO_2_, carbonate, and bicarbonate through buffer reactions. These effects are, of course, important to consider in the simulation of devices for the electrochemical CO_2_RR.^17,26,27^ However, due to the complex 2-D geometry of the simulated system, convergence is challenging when a complete treatment of all ions in the electrolyte is employed due to the immense increase in the number of degrees of freedom calculated when the ionic species are included, as well as the sharp concentration gradients generated by the nonlinear buffer source terms. Therefore, because the primary aim of the study is to explore membrane hydration in these systems, a secondary current distribution, which still enables the capture of hydration-dependent charge transport and electro-osmosis on bulk water transport, is adequate to describe the essential physics.

When using the secondary current distribution, the ionic current in the electrolyte is calculated by Ohm's law,

1 *i*_l_ = −*σ*M∇*ϕ*_l_,

where *σ*_M_ is the ionic conductivity of the membrane (see ESI Methods† for definition of transport properties in the model), *ϕ*_l_ is the ionic potential, and *i*_l_ is the ionic current density.

The electronic current in the porous electrodes is defined also through Ohm's law,

2 *i*_s_ = −*σ*_s_∇*ϕ*_s_

where *σ*_s_ is the electronic conductivity of the solid phase of the porous electrodes, *ϕ*_s_ is the electrostatic potential, and *i*_s_ is the electronic current density.

The ionic and electrostatic potentials are related by Tafel kinetics for the CO_2_RR and hydrogen evolution reaction (HER) at the cathode, as well as the acidic or alkaline oxygen evolution reaction (OER) at the anode. Employing Tafel kinetics assumes all reactions are irreversible, which is likely true for the high applied potentials modeled. Standard potentials are evaluated at a pH of pH_0_ = 8.14.3
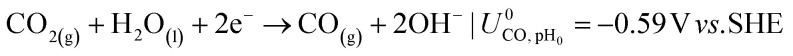
4
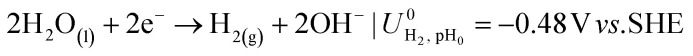
5
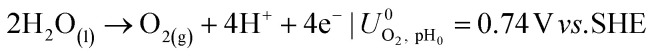
6
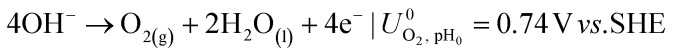


For cathodic reactions, the kinetics are modeled using the following expression:7
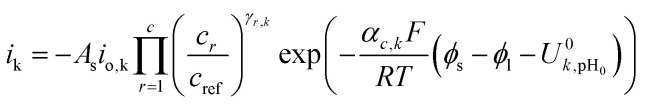


For anodic reactions, the kinetics are modeled using the following expression:8
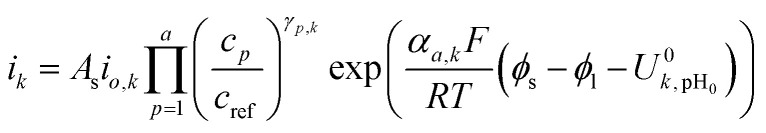
In the Tafel expression, *A*_s_ is the surface area to volume ratio of the porous electrode, *i*_*o*,*k*_ is the exchange current density of heterogeneous reaction *k, γ*_*i*,*k*_ is the rate order of reaction *k* with respect to species *i*. The subscript *c* denotes a cathodic reaction, and subscript *r* denotes the reactant species in a given cathodic reaction. The subscript *a* denotes an anodic reaction, and subscript *p* denotes reactant species in a given anodic reaction. *c*_ref_ is a reference concentration set to the concentration at unit activity (1 M). *α*_a/c,*k*_ is the transfer coefficient of the given anodic or cathodic half reaction, *R* is the ideal gas constant, *F* is Faraday's constant, *T* is the temperature (assumed to be a constant 298 K), and *U*_*k*,pH_0__0 is the equilibrium potential of the given half reaction evaluated at pH_0_.

The concentration of CO_2_ in the electrolyte is determined using Henry's Law,

9 *c*_CO_2_,l_ = *p*_CO_2_,g_*H*_CO_2__

where *p*_CO_2_,g_ is the partial pressure of CO_2_ in the gas phase, and *H*_CO_2__ is the Henry's Law constant for CO_2_ dissolution in water.

### Concentrated species transport in the gas phase

The gas phase contains CO_2_, H_2_O, H_2_, CO, N_2_, and O_2_. The mole fractions in the gas phase are calculated from,

10 ∇·*N*_*i*_ = *R*_CT,*i*_ + *R*_PT,*i*_

The flux, *N*_*i*_ is defined by the following relationship, 11
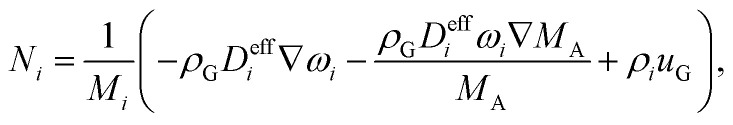
where *u*_G_ is the bulk velocity of the gaseous mixture, *M*_A_ is the average molecular weight of the mixture, and *ρ*_G_ is the gaseous mixture density. Additionally, *M*_*i*_ is the molar mass of species *i*, *ω*_*i*_ is the mass fraction of species *i*, and *D*^eff^_*i*_ is the effective diffusion coefficient of species *i* in the gaseous mixture.

### Water transport in the ionomer phase

The molar flux of water, *N*_w_, through the ionomer occurs by two mechanisms: diffusion and electro-osmosis.12
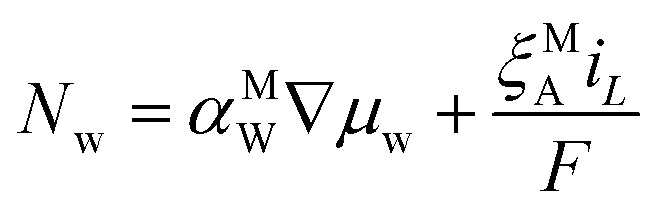
where *α*^eff,M^_w_ is the water transport coefficient in the AEM, which is itself a function of the water activity,^28^ and *ξ*^M^_A_ is the electro-osmotic coefficient. Anions carry current in the AEM, so the sign is negative because water flows in the direction opposite to the current density. The chemical potential of water, *μ*_w_, is defined by13
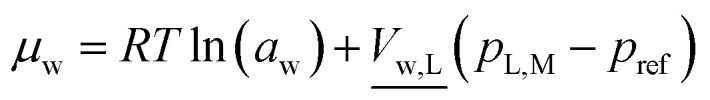
where 
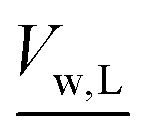
 is the molar volume of liquid water, and *a*_w_ = *p*_v_/*p*^vap^_w_ is the activity of water vapor referenced to its vapor pressure. The water chemical potential is solved for by solving the mole balance:

14 ∇·*N*_w_ = *R*_CT,i_ + *R*_PT,i_

It is important to note that the water in both the GDE and membrane, as modeled, are within ionomer environments; in other words, the simulated GDE possesses Ag catalyst particles suspended within an anion-exchange ionomer. This is crucial, because the ionomer in the GDE plays an important role in water management for the system. The ionomer is crucial in the case of excess water, because it mitigates flooding and enables improved transport of the reactant CO_2_ to the catalyst sites.^26^ It is also notable that the presence of the ionomer will change the local microenvironment in the catalyst layer, particularly, with respect to the local CO_2_ to H_2_O ratio due to the lower water availability in the anion-exchange ionomer compared to the liquid water present in a flooded GDE catalyst layer.^29^ However, at higher current densities, the reduced water availability in the catalyst layer ionomer can become limiting. Therefore, for the case of simulation, we choose to model the system with an ionomer in the catalyst layer because it possesses the most opportune microenvironment for potential application in CO_2_RR devices, as well as the most severe case of potential catalyst layer dry-out.

### Bulk fluid flow in porous media

The gas and liquid pressures in the porous media domains (PTLs and CLs) are calculated using mass conservation and Darcy's Law as follows.

15 ∇·*ρ*_p_*u*_p_ = *Q*_p_

16
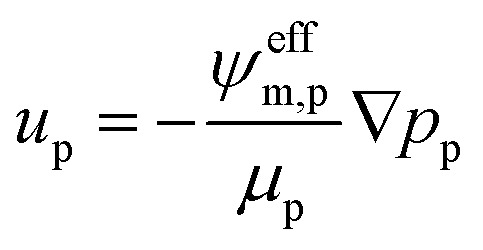
In the above expressions, *u*_p_ is the mass-averaged velocity field of phase *p*, *ψ*^eff^_m,p_ is the effective permeability of phase *p* in a porous medium, *m*, and *p*_p_ is the bulk pressure of phase *p*. *μ*_p_ and *ρ*_p_ are the viscosity and density of phase *p*, respectively.

### Boundary conditions

The boundary conditions are detailed in Table 1. At the leftmost boundary of the anodic PTL (the interface of the anode channel (CH) and the PTL), the potential is set to the applied cell potential. At this boundary, the gas pressure is set to 1 bar. The liquid-phase Darcy's Law boundary is defined as follows: if the liquid pressure, *p*_L_, is less than or equal to the gas pressure, then a no-flux boundary condition is applied. Conversely, if the liquid pressure exceeds the gas pressure, there is an outward flux of liquid water with an arbitrarily high mass-transfer coefficient (*k*_MT_ = 1 kg m^−2^ s^−1^) to maintain the pressure balance at the boundary. At the interface of the cathode CH and PTL, the gas and liquid pressures remain the same as for the anode side, but the solid-phase potential is now set to ground (0 V). At the interface of the PTL and the CLs, the ionomer water flux and the ionic flux are both set to 0. Lastly, at the interface of the internal channel and the membrane, the chemical potential of water in the membrane is set to 0 (*i.e.*, the liquid pressure in the membrane is set to its reference pressure of 1 bar).

Boundary conditions for the continuum simulation

**Table:** 

	Anode CH|PTL	Cathode CH|PTL
*ϕ*_S_	*V*_cell_	0 V
*p*_G_	1 bar	
*p*_L_	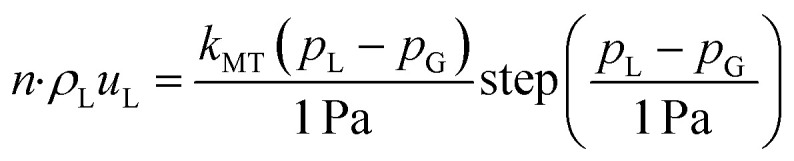

**Table:** 

	Anode PTL|CL	Cathode PTL|CL	Internal CH|Mem
*ϕ*_L_	∇*ϕ*L = 0	∇*ϕ*_L_ = 0	
*μ*_0_	∇·*N*_w_ = 0	∇·*N*_w_ = 0	*μ*_0_ = 0

### Numerical methods

All governing equations were solved using COMSOL Multiphysics 5.6 software with the PARDISO solver with a relative tolerance of 0.001. The modeling domain was adaptively refined, with element size decreased sharply near domain boundaries to capture gradients in the simulated quantities.

## Experimental

### Membrane fabrication

For the preparation of the AEM, an imidazolium-functionalized poly(phenylene oxide) (ImPPO) polymer with 13% of functionalized methyl pendants was synthesized as described elsewhere.^30^ To fabricate a membrane with internal microchannels, a mold with these channels was first milled in polyether ether ketone (PEEK). Afterwards, a positive mold was made in polydimethylsiloxane (PDMS). 0.26 g of ImPPO was then dissolved in ∼4.5 mL of NMP, the solution was cast in the PDMS mold, and left in the oven at 70 °C for ∼36 h until the solvent had completely evaporated. This resulted in a membrane material with the profiled channels (Fig. 2b). The inlet and outlet holes were reamed into the sides (Fig. 1). To enclose the channels, a 40 μm thin film of ImPPO without channels was attached with a minimal amount of NMP. For additional tests and comparison, two membranes without channels were fabricated: a 32 μm-thick membrane, and a membrane with the same thickness (170 μm) as the one with internal microchannels.

### Electrochemical cell tests for flat membrane

The ImPPO membrane conductivity and CO_2_ reduction characteristics was tested using a 5 cm^2^ electrolyzer from Dioxide Materials, with 0.1 M KOH anolyte and a humidified CO_2_ stream at the cathode. The cathode was a carbon paper GDE (Sigracet 38 BC) sputtered with 100 nm-thick silver layer, and the anode was a nickel foam electrode. Using an IviumStat.f potentiostat, a current of 100 mA cm^−2^ was applied for 30 minutes, while measuring the voltage. The gas outlet was analyzed using a gas chromatograph (CompactGC, Interscience).

### Electrochemical cell tests for membrane with internal microchannels

A 2.25 cm^2^ electrolyzer was custom-made with an inlet and outlet for the membrane's internal microchannels. It was first used to measure the membrane resistance with deionized (DI) water or different electrolyte concentrations inside the channels using the fast current-interrupt method. On the two sides of the cell, 0.1 M KHCO_3_ was used as catholyte and anolyte. Two micro reference electrodes (Ag/AgCl) from Alvatek were placed near the inlets of the catholyte and anolyte in the cell and a current of 5 mA cm^−2^ was applied until a stable voltage was observed (Fig. S4†). By interrupting the current, the drop in voltage corresponding to the ohmic resistance was immediately observed. The electrolyte inside the channels was pumped at 0.2 mL min^−1^ using a syringe pump and the anolyte and catholyte were flowed at 1.3 mL min^−1^. The concentration of KHCO_3_ inside the channels was varied between 0 and 0.5 M.

For the electrolysis experiments, a humidified (89–95% humidity) CO_2_ gas stream was used on the cathode side, while the anolyte was either 0.1 M KOH or a humidified N_2_ stream. The liquid flow rates were the same as those used in the resistance experiments and the gas flow rates were set at 50 sccm. Initially, a voltage of 3 V was applied to the cell for 30 minutes in order to ensure that the membrane was equilibrated with the ions produced during CO_2_ electrolysis, such as HCO_3_^−^ and CO_3_^2−^. The voltage was then swept linearly at 0.1 V s^−1^ up to 3.75 V and the current was recorded continuously.

For the K^+^ crossover experiments, a constant current of 5 mA cm^−2^ was applied for 70 minutes. A sample was taken from the electrolyte in the membrane microchannels before and after the experiment, and the cathode GDE was immersed in acidified water with 15% isopropanol and stirred overnight. Both solutions were then filtered and analyzed using ion chromatography (Metrohm 881 Compact IC Pro). This method allows the estimation of the amount of K^+^ ions which have crossed from the anolyte to the cathode surface.

## Results and discussion

### Macroscale simulations

#### Effect of internal channel geometry and location

**Fig. 3:**
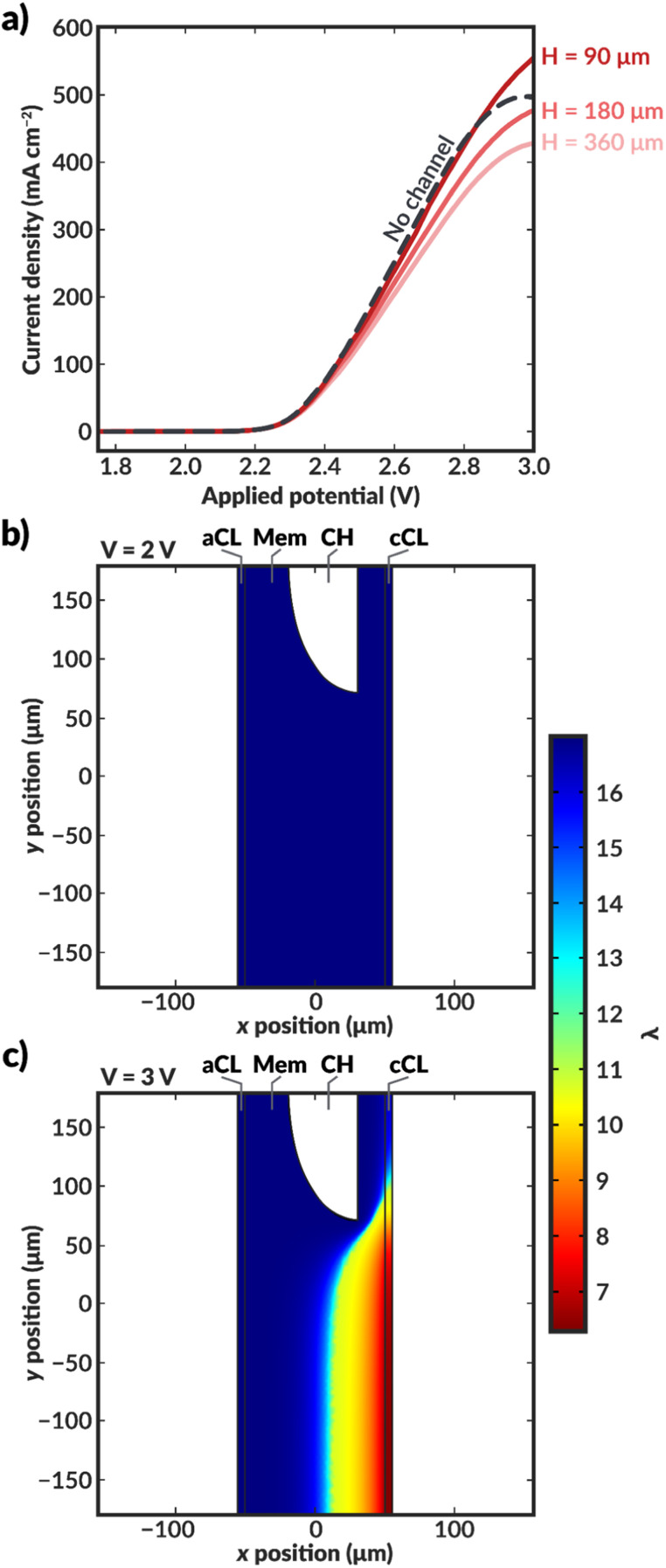
(a) Simulated polarization curves for bilayer AEM-MEA systems (red solid lines) with various distances between the centers of the internal microchannels (see schematic in Fig. 2 for definition of *H*), as well as for an AEM-MEA with no internal channel (black dashed line). Simulated water content distribution within the MEA for a bilayer MEA with *H* = 360 μm at applied voltages of (b) 2 V and (c) 3 V. For these simulations, the aspect ratio of *H* to the channel height (*H*_chan_) is constant. If *H* is scaled by 0.5, *H*_chan_ is similarly scaled by 0.5. The thickness of the channel is constant. The simulated water content and intermediate applied potential of 2.5 V can be found in ESI Fig. S6.†

To explore how the implementation of an internal membrane channel impacts the performance of a vapor-fed MEA CO_2_ electrolyzer, the local water content and current densities were simulated for various applied potentials in a macroscale model of the device. Considering the low conductivity of the DI-filled internal channel and the expected improved hydration, the size and spacing of the internal channels are important parameters. As shown in Fig. 3a, when a channel with half-distance of the channel centers (*H*) of 360 μm is incorporated into the membrane (*i.e.*, 720 μm spacing, 218 μm channel height), the current density is lower than that of a device with no channel at potentials greater than approximately 2.4 V. This reduction is a result of the increased ohmic resistance incurred due to loss of direct ionic pathways between the anode and cathode when a DI water-filled channel is incorporated into the cell. However, the hydration of the membrane is substantially improved (Fig. 3b–c and S7†), thus there is a tradeoff between ionic path tortuosity and membrane hydration. Notably, sections of the cathode catalyst layer (cCL) closest to the internal channel are fully hydrated, with the AEM being fully liquid equilibrated with a water content (*λ*) of 17, where the water content is defined to be the moles of water in the membrane or CL ionomer domain per moles of fixed charge.^31^ This improvement in the membrane hydration could potentially lead to longer AEM lifetimes.^32,33^

When *H* is decreased proportionally along with the channel height (*H*_chan_) (*i.e.*, the channel spacing is decreased), the polarization characteristics improve (Fig. 3a). When the channel spacing decreases by a factor of four (*H* = 90 *vs. H* = 360 μm), the MEA can achieve a substantial 100 mA cm^−2^ enhancement in the current density at an applied potential of 3 V. This enhancement results from improved hydration reducing the ohmic losses incurred in the cell. For this case, the improved ionic conductivity through the membrane due to increased hydration around the channels outweigh the low conductivity of the DI water in the channels themselves. The water content distributions for the no channel, along with the *H* = 90 and 180 μm cases can be found in ESI Fig. S7–S9.† As simulated, for a given applied potential, bilayer AEMs with lower channel spacings possess larger, more uniform water content values.

To further highlight the role of hydration in these ionomers, it is instructive to calculate the average tortuosity of these modified AEMs. These tortuosities are calculated using power loss analysis^34,35^ for the fully hydrated AEM at low applied potentials to calculate an effective conductivity of the AEM that accounts for the increased average path length required to traverse around the water channel (see ESI†). The ratio of the conductivity of the membrane without the water channels to the effective conductivity of the membrane with the water channels represents the increase in the tortuosity of the ion conduction pathways. The tortuosities of all three AEMs with channels are greater than one, meaning that the ionic conductivities through these AEMs should be lower than that of an AEM with no channels due to the increase in the average path length at the same level of hydration. This increased tortuosity may explain the reduced current density supported by the bilayer AEMS with *H* = 180 and 360 μm, despite their higher hydration than the AEM without channels. However, for the bilayer AEM with *H* = 90 μm, the increased hydration overcomes the losses due to increased tortuosity, and the AEM with channels supports a higher current density at 3 V than the AEM without channels.

**Fig. 4:**
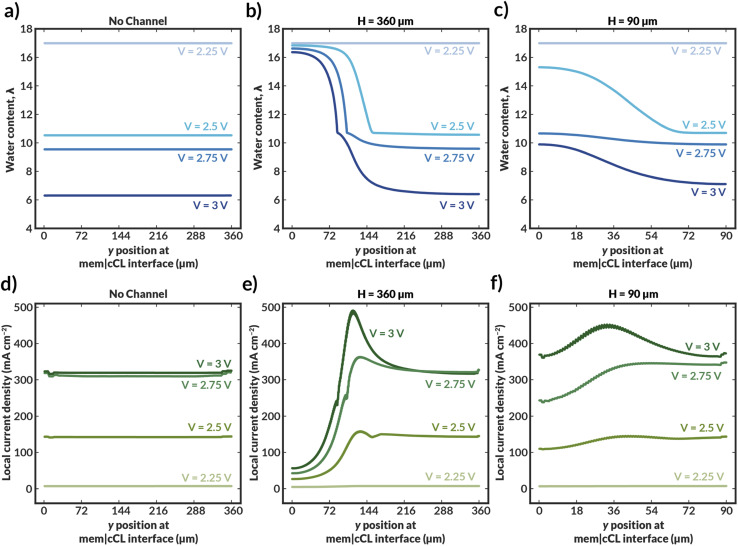
(a–c) Local water content of the ionomer within the cathodic CL averaged across thickness of the CL for varying channel geometries and spacings. (d–f) Local current density within the cathodic CL averaged across thickness of the CL for varying channel geometries and spacings.

Further insight into how the internal channels impact local water content in the CO_2_RR catalyst environment can be gained by exploring the water content and local current density distributions within the CLs for the various simulated bilayer AEMs (Fig. 4). The water content in the cathode CL is critical to simulate, as it represents the availability of water to the catalyst to perform CO_2_RR. Previous work has demonstrated that the water content of the cathode CL impacts both the activity and selectivity of the CO_2_RR.^17,36^ Additionally, work by Disch *et al.* has used neutron-imaging to experimentally demonstrate that the loss of water content is prevalent in Ag-AEMEAs performing the reduction of CO_2_ to CO, and the simulated water content profiles for the no-channel case are consistent with the trends shown experimentally, wherein upon increases in current density, the water content near the membrane-GDE interface is substantially reduced.^37^ Interestingly, the average water content at the CL at a given applied potential is reduced at lower channel spacings, particularly around the channels themselves (Fig. 4b and c). This behavior can be rationalized as follows. For the bilayer AEM with *H* = 360 μm, the internal channels are relatively large, and ionic current does not readily pass around the channel, essentially making a portion of the catalyst layer inactive for CO_2_RR, as evidenced by the low to negligible local current densities observed near the internal channel (Fig. 4e). Therefore, a portion of the CL near the internal channel remains well hydrated because water is not consumed by the CO_2_RR. Conversely, the CL adjacent to channels with *H* = 90 μm remains active for CO_2_RR because the ionic transport pathways are less impeded around the smaller channels and ionic current can pass through the bulk AEM. While the improved CL utilization is promising, high water consumption is observed due to the CO_2_RR and water content is reduced as the applied potential is increased. Nonetheless, for both bilayer AEMs with *H* = 90 and 360 μm, the water content at a given applied potential within the CL is markedly higher than in the AEM with no channel, and in the *H* = 90 μm case, the local current densities are also enhanced due to improved CL utilization. The analysis of these water content and current density profiles reveals two challenges with these materials: the low conductivity of the internal water channels can lead to poor CL utilization, and water consumption in the CL remains a challenge even at low channel spacings. A potential solution to these issues is to use the thinnest possible channel in the in-plane direction to mitigate loss of active area, and to position the channel as close to the CL as possible to better maintain hydration in the vicinity of water consumption.

**Fig. 5:**
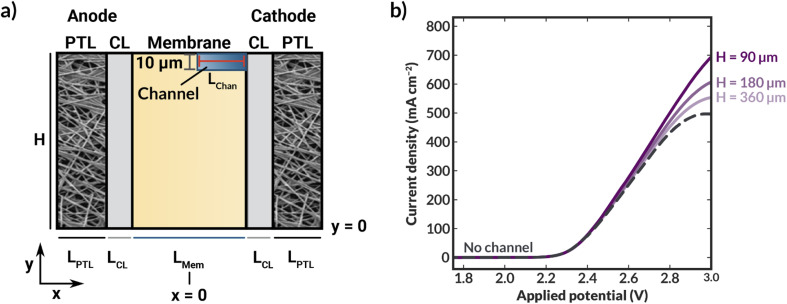
(a) Schematic of 2-D geometry employed to model an AEM with the internal channel placed adjacent to the cathode CL. Contrary to the prior simulations where the channel height was scaled proportionally to the channel spacing, the channel height was fixed at 10 μm in these simulations, representing the smallest height reasonably manufacturable. (b) Polarization curves of bilayer AEMs with cathode-adjacent channels of varying channel spacings.

To confirm these theorized impacts of variations in the channel size and location, additional geometries were simulated. For these models, *H*_chan_ was fixed to 10 μm (representing a small channel that could be reasonably be manufactured with techniques such as additive manufacturing^38–40^) and *H* was varied independently of *H*_chan_, with the channel positioned directly adjacent to the CL. Additionally, due to challenges with convergence with the curved channel implemented for previous simulations, the geometry was made rectangular while retaining the same *L*_chan_ (Fig. 5a). Following the hydration patterns in case of curved internal channels (*e.g.* (Fig. 3c)), we expect little effect on the current density when changing to a rectangular channel shape. As shown in the simulated polarization curves in Fig. 5b, this implementation of the channel exhibits higher current density at all channel spacings (*H* = 90, 180, and 360 μm) than in the prior implementation or for the AEM with no channel. Because the channel is so thin, ion transport to the CL is blocked very little, which minimizes the locally low current density as was the case in Fig. 4e and f. In addition, positioning the channel directly adjacent to the CL maintains better AEM hydration than the implementation wherein there is a polymer layer between the channel at the CL (*cf*., Fig. 2). Although the fabrication of such a thin channel is challenging and its positioning directly at the interface of the AEM and the CL may pose structural challenges, the simulations presented herein demonstrate the utility of geometric levers that can control the activity and performance of CO_2_RR devices employing *operando* water management strategies.

#### Effect of internal channel electrolyte conductivity

**Fig. 6:**
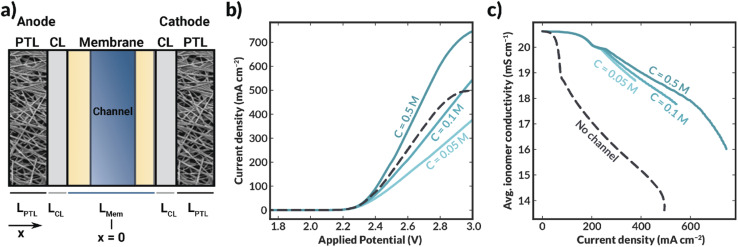
(a) Schematic of 1-D geometry modeled (channel thickness of 50 μm) to determine the impact of channel electrolyte concentration on cell performance. (b) Current–voltage characteristics of bilayer AEM cells with an internal channel with varying KHCO_3_ concentration. (c) Average ionomer conductivity within the bilayer AEM with varying KHCO_3_ concentration as a function of total cell current density.

We also simulated the case in which the internal channel is extending through the full height of the membrane. Such a case is relatively straightforward to manufacture by, for example, using spacers in between thin flat membrane layers. Moreover, it collapses the computationally costly 2-D model to a 1-D model as shown schematically in Fig. 6a.

Simulations demonstrate that, as the concentration (and, consequently, conductivity) of supporting electrolyte within the channel increases, the cell current density also increases (Fig. 6b). Additionally, the membrane is more highly hydrated (as evidenced by the average polymer conductivity, Fig. 6c) in all cases where the channel is included than in the case where no channel is included. Interestingly, when the channel contains 0.1 or 0.05 M KHCO_3_, the cell largely exhibited lower current densities than the no-channel case at potentials greater than approximately 2.3 V (Fig. 6b), despite the observed increase in conductivity of the ionomer domains when the channel was included (*cf.*, Fig. 6c). In these systems, performance is limited by the low conductivity of the aqueous electrolyte in the channel.

## Experimental results

### Effect of internal channel implementation and composition on cell resistance and performance

The previously synthesized imidazolium-functionalized poly(phenylene oxide) was cast into a 32 μm-thick membrane to test its conductivity under electrolysis conditions. As shown in Fig. S2,† a CO_2_ electrolyzer containing this membrane exhibited a stable cell voltage of approximately 3.6 V when a current of 100 mA cm^−2^ was applied, with an average FE for CO of 74%. While this device incorporating ImPPO does not outperform state-of-the-art membranes such as Sustainion and PiperION in terms of conductivity, they greatly outperform devices incorporating other commercial membranes like Fumasep and Selemion.^14,41^

**Fig. 7:**
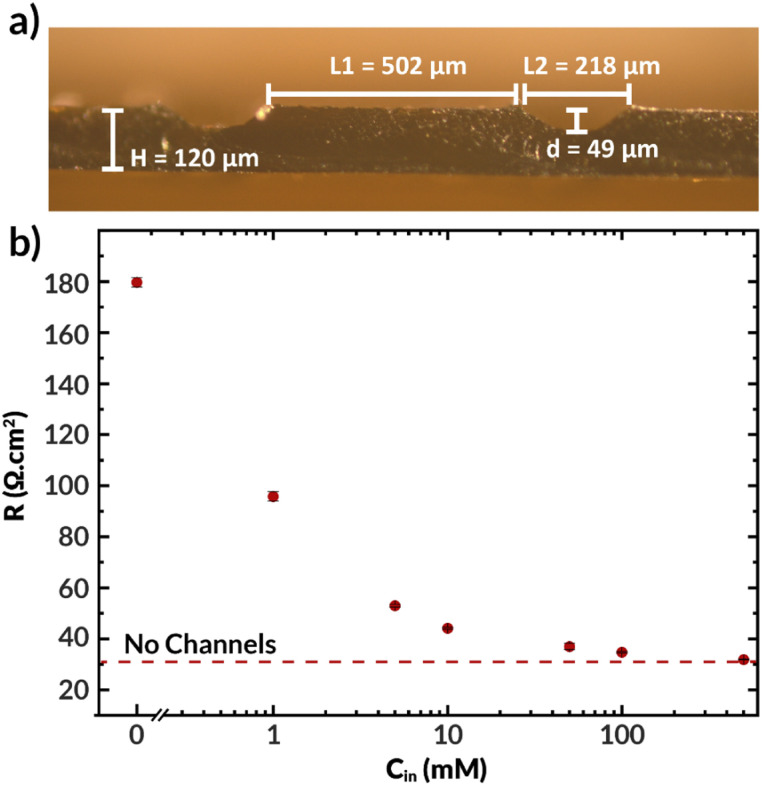
(a) Optical micrograph of ImPPO layer with channels in hydrated state. For the experiments, a thin ImPPO layer was attached (not shown) to cover the top side of the channels. (b) Ionic resistance of the membrane *vs.* the concentration of KHCO_3_ in the microchannels (*C*_in_). The concentration outside of the membrane was 0.1 M KHCO_3_ at either side.

The microchannels resulting from casting the AEM as described previously are shown in Fig. 7a. This film was subsequently joined with another thin AEM layer using a minimal amount of NMP to create a bilayer membrane with internal microchannels. The resulting channels were 59 μm deep, 216 μm high, and were spaced 502 μm apart. The center-to-center spacing (502 + 216 = 718 μm) is approximately the same as the geometry of the first simulated AEM with *H*_chan_ = 360 μm (2 × 360 = 720 μm spacing). The total hydrated membrane thickness was ∼170 μm, which is significantly thicker than the 25–50 μm thickness of state of the art membranes.^14,18^ Improved fabrication methods, such as stereolithography or techniques involving additives^31–33^ may be needed to achieve such dimensions with the presence of internal microchannels. Nonetheless, this membrane facilitates the experimental study of how the electrolyte concentration inside the channels influences the membrane properties and observables within the electrolysis process, such as the current density, applied voltage, and salt crossover/deposition.

Fig. 7b displays the ionic resistance of the membrane as a function of the KHCO_3_ concentration in the internal microchannels. Consistent with the simulated results shown above, the ionic resistance greatly increases with decreasing electrolyte concentration in the channels. Only at the highest tested concentration of 0.5 M, the membrane exhibited approximately the same conductivity as a membrane of equal thickness without channels. Therefore, in fully hydrated conditions, the presence of channels does not improve the membrane conductivity. Future work should aim to quantify the ohmic resistance of the bilayer AEM at high current densities, potentially *via in situ* high-frequency resistance measurements or the current interrupt method.^42^ Additionally, future work should seek to use *in situ* techniques of water management, such as neutron imaging,^43^ X-ray tomography,^44^ and magnetic resonance imaging^45^ that have been used to measure water content in fuel cells to better relate changes in conductivity to changes in water content.^46^

It is important to note that all the observed resistance values are higher than expected, with typical values for membranes of this thickness being between 4 to 10 Ω cm^2^,^47,48^ and that the resistance is highly sensitive to the electrolyte concentration in the internal channels. Although electrolyte concentration is known to affect polymer conductivity,^49^ the internal channels are small relative to the distance between them, and hence the external electrolyte should not dominate the membrane's conductivity. The thin (32 μm) AEM without channels had an ionic resistance of 6.2 Ω cm^2^, which means that the polymer's bicarbonate conductivity should be improved. This can be achieved by, for example, tuning the polymer degree of functionalization.^30^

**Fig. 8:**
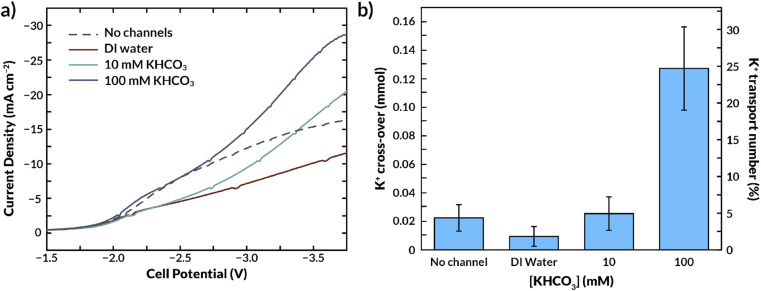
(a) Galvanostatic linear sweep experiments with different electrolyte concentrations inside the AEM channels. (b) K^+^ crossover to the cathode side in function of the KHCO_3_ concentration inside the microchannels of the AEM.

Carbon dioxide reduction experiments were performed to validate the effect of internal channels on the cell's energy efficiency and ion crossover. Fig. 8a and S9† show the current response to a linear voltage sweep with 0.1 M KOH as an anolyte and in a full MEA configuration, respectively, as a function of the concentration of KHCO_3_ in the microchannels.

Fig. 8a and S11† show that for this channel geometry, flowing DI water in the internal microchannels reduces the current density at a fixed voltage. This result is due to the very low conductivity of the DI water, which increases the overall ohmic resistance while also reducing CL activity as observed in the simulations. Employing a 10 mM KHCO_3_ concentration in the microchannels substantially increases conductivity with respect to DI water when operating in aqueous solutions at both sides (see Fig. 7b), and this trend is also observed when fed with humidified CO_2_ gas (Fig. 8a). The required cell potential is less negative compared to that of the standard AEM (*i.e.*, without microchannels) beyond a current density of ∼15 mA cm^−2^. This result further highlights that the role of the internal channels is more pronounced at higher current densities. A 100 mM KHCO_3_ concentration inside the microchannels increases the current density even further, well beyond that of the standard AEM (Fig. 8a). Because it has previously been established that the bilayer AEM is not more conductive than the standard AEM under fully hydrated conditions (Fig. 7), it is likely that increased membrane hydration under the applied potential is critical to this result. Unfortunately, the maximum achievable current density was 30 mA cm^−2^ due to the membrane thickness and conductivity. Nonetheless, we expect that at higher current densities the (de)hydration effect would be even more prominent. This result suggests that membrane hydration could limit CO_2_ electrolysis efficiency as current densities are increased.

### Effect of internal channels on salt crossover

Because K^+^ ion availability has also been reported to increase the activity of the catalyst layer, the microchannels could improve the selectivity of the catalyst toward CO by supplying cations.^50–52^ Conversely, an excess supply of K^+^ can lead to salt deposition and deactivation of the catalyst.^17^ Thus, MEAs for CO_2_RR will need to manage salt fluxes.^34^Fig. 8b shows the K^+^ crossover to the cathode GDE as a function of the KHCO_3_ concentration in the internal microchannels. Consistent with prior CO_2_RR literature,^15,17,23^ a significant amount of K^+^ crosses over through the AEM from the anolyte (100 mM KOH) in the absence of internal channels. Interestingly, the presence of an internal microchannel filled with DI water reduces the amount of K^+^ that reaches the cathode. The K^+^ concentration of the internal channel feed increased at time progressed during the experiment, implying K^+^ is captured in the internal channel and washed away along with the outlet water.

A dilute solution in the internal microchannels is also likely to make the surrounding membrane material more selective to counter-ions, as the Donnan exclusion is most effective at low electrolyte concentrations.^25^ The classical Donnan model predicts that the ratio between co-ions (K^+^) and counter-ions (HCO_3_^−^, CO_3_^−^ or OH^−^) in the membrane decreases when the KHCO_3_ concentration in the aqueous phase decreases (see ESI eqn (S22)†). Therefore, a low KHCO_3_ concentration in the internal channel not only reduces the driving force for K^+^ crossover to the CL, but also causes an increased selectivity due to the relatively low sorption coefficient of K^+^ in the membrane. Hence, this would imply that the total K^+^ crossover (to cathode and internal feed together) would be lower compared to having a membrane without internal channels. We calculate from eqn (S22)† that changing the KHCO_3_ concentration from 100 mM to 10 mM, assuming a membrane charge density of 5 M, the potassium concentration in the membrane decreases by a factor 100. Although the effect of increased selectivity cannot be distinguished from the ions being removed by the internal feed at this scale of experiments (the channel volumes do not allow for an accurate mass balance), it is clear that the actual decrease is crossover is less extreme (0.13 at 100 mM to 0.025 at 10 mM). We conclude that the distance between the microchannels is too large to fully leverage the effect of increased selectivity.

The internal microchannel feed concentration was also expected to have an influence on the FE for CO, since literature shows that the presence of K^+^ in the catalyst layer also increases the selectivity of the process.^24,53^ However, the FE remained constant (∼58%, Table S3†), since such changes in selectivity cannot be observed in the current density range in our experiments.

Utilizing a 10 mM KHCO_3_ electrolyte concentration in the microchannel had an insignificant impact on the K^+^ crossover compared to the K^+^ crossover through a membrane without internal channels. However, substantial salt deposition on the cathode was observed when 100 mM KHCO_3_ was used. Therefore, there is a balance between the current density that can be feasibly achieved in this system and the salt deposition, which both depend on the electrolyte concentration in the microchannels. Our results suggest that electrolyte concentrations up to 10 mM KHCO_3_ could be advantageous since they promote membrane hydration at high current densities, improve its conductivity, and do not contribute significantly to salt deposition.

This concept of incorporating microchannels into an AEM exhibits great potential, but some practical challenges remain. These challenges are primarily linked membrane thinness requirements and the need for reduced spacing of the channels within the AEM to enhance conductivity. Altogether, for this system to be feasible, the channels must be fabricated with a channel height on the order of 10 μm. Although it is possible to fabricate such a channel, the pressure drop would be substantial (∼1.7 bar per cm of channel at 50 μL min^−1^). However, the small radii of such channels would make them ideal for leveraging capillary forces by connecting the channels to a water or electrolyte tank.^19^ In this case, if any water were consumed, it would be replenished without requiring pumping. Similarly, recent work has demonstrated 3-D printed cellular fluidics that provide programmable management of gas and liquid flows *via* capillary action.^39^

## Conclusions

In summary, we have simulated and experimentally quantified the transport properties of an AEM with internal microchannels for CO_2_ electrolysis. Both our simulations and experiments indicate that membrane hydration is a major challenge in AEMs due to electro-osmotic fluxes and catalytic consumption of water in the CO_2_RR. The presence of internal microchannels helps to maintain membrane hydration, which can increase the current density. These channels, however, can impede ionic pathways, especially if a low conductivity fluid is flowing through, effectively deactivating portions of the catalyst blocked by the channels. We conclude that internal channels should be on the order of 10 μm in size. Furthermore, they should be positioned close to the cathode surface to better hydrate and provide water for the catalytic CO_2_RR that occurs within the cathode CL. Modeling reveals that the distance between channels should not exceed 90 μm, and that a membrane with channels of optimal geometry can increase the current density by up to 40% compared to a standard AEM. It is important to note that this value will likely change with the material properties of the AEM(*e.g.*, thickness, ion exchange capacity, electro-osmotic coefficient, *etc.*), and further work should aim to design optimized ionomer chemistry for this specialized application. Modeling and experimental results have shown that current density increases with increasing electrolyte concentration inside the channels. Furthermore, experimentally, a membrane with microchannels (*H*_chan_ = 360 μm, *L*_chan_ = 50 μm) containing 0.1 M KHCO_3_ electrolyte exhibited a significant increase in current density compared to a membrane without these microchannels. Since the presence of channels does not make the overall membrane more conductive in fully hydrated state, this result provides further evidence for the importance of water management in an electrolyzer. Lastly, we have also observed experimentally that electrolyte concentrations in the channels higher than 10 mM promote K^+^ cross-over to the cathode. Nonetheless, concentrations lower than 10 mM inside the channels do not meaningfully contribute to salt deposition, but promote membrane hydration and increasing its overall conductivity.

While there are still substantial challenges with regard to the fabrication and implementation of these bilayer AEMs, particularly with regard to making channels small and close enough such that they result in substantive gains in performance. Recent work in additive manufacturing provides great promise for ameliorating these concerns by enabling the generation of cellular fluidics with well-defined and geometrically controlled capillary flow in unit cells,^39^ and capillary fluidics may enable the implementation of internal microchannels without induced flow. This work could initiate the development of a new class of materials with *in operando* water management that can alleviate dehydration in MEA CO_2_RR devices, and could potentially be critical in the implementation of electrolyzer technology at scale.

## Conflicts of interest

There are no conflicts to declare.

## Supplementary Material

SE-006-D2SE00858K-s001
