# Supplementary material for: Anion-exchange membranes with internal microchannels for water control in CO2 electrolysis
Source: Sustain Energy Fuels. 2022 Sep 28;6(22):5077–88. doi: 10.1039/d2se00858k (PMC9642111; doi:10.1039/d2se00858k)
Supplement: SE-006-D2SE00858K-s001 [file SE-006-D2SE00858K-s001.pdf]

Electronic Supplementary Material (ESI) for Sustainable Energy & Fuels.
This journal is © The Royal Society of Chemistry 2022

Supporting Information for: Anion-exchange membranes with internal microchannels for water control in CO_2 electrolysis

Kostadin V. Petrov[†1], Justin C. Bui[†2,3], Lorenz Baumgartner[1], Lien-Chun Weng[2,3], Sarah M. Dischinger[3], David M. Larson[3], Daniel J. Miller,[3] Adam Z. Weber[3], David A. Vermaas[1]

[1] *Department of Chemical Engineering, Delft University of Technology, 2629 HZ Delft, The Netherlands*

[2] *Department of Chemical Engineering, University of California, Berkeley, California 94720-1462, USA*

[3] *Joint Center for Artificial Photosynthesis, Lawrence Berkeley National Laboratory, California, 94720-1462, USA*

[†] Contributed equally

Corresponding Authors: azweber@lbl.gov, D.A.Vermaas@tudelft.nl

Supplementary Computational Methods

Definition of Transport Properties:

In the ionomer domains, the conductivity of the electrolyte is

$$\kappa_M = (1 - S_M)\kappa_V + \kappa_L S_M \tag{1}$$

In the above expression, κ_L is the conductivity of a liquid-equilibrated AEM, which is set to a constant value of 20.6 mS cm^{-1} (See **Table S1** for a summary of parameter values used in the simulation).[1] κ_V is the conductivity of a vapor-equilibrated AEM, which is a function of the vapor activity (a_w) by

$$\kappa_V = 0.003 exp(8.1432 a_w) \tag{2}$$

S_M is defined by an empirical relationship roughly related to the interior surface energies and water-phase network.[18] When S_M is 1, the ionomer is fully liquid equilibrated, when S_M is 0, the ionomer is fully vapor equilibrated.

σ_S of the porous electrode is defined to be 220 S m^{-1} for the diffusion medium, and 100 S m^{-1} for the catalyst-layer domains.

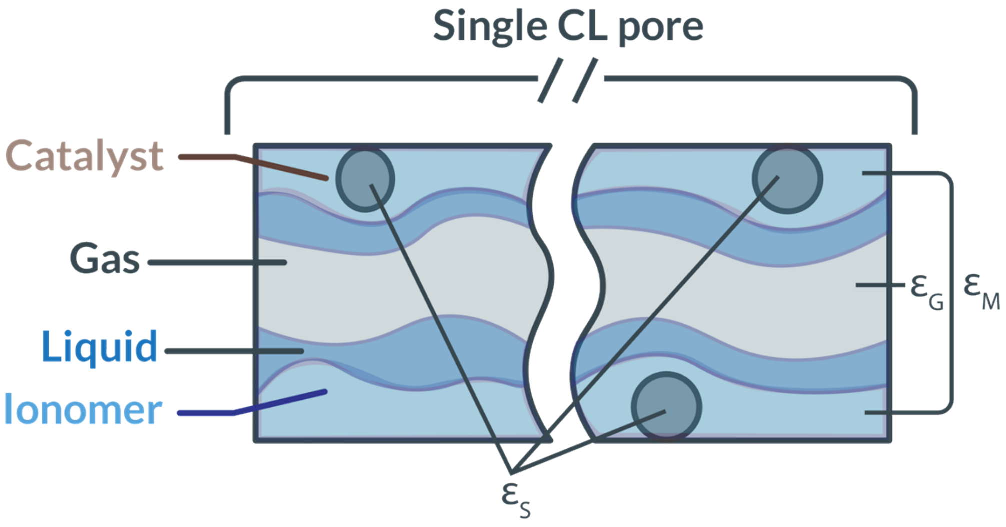

Figure S1 – Schematic of a single pore within the porous catalyst layer in the electrochemical model. As simulated, the porous catalyst layer is assumed to be a homogeneous continuum of CL pores with volumes defined as shown above.

Lastly, all conductivities in the porous-electrode domains (shown schematically in **Figure S1**) are corrected for tortuosity and porosity using the Bruggeman correlation,

$$\kappa^{eff} = \varepsilon_p^{1.5}\kappa \tag{3}$$

where ε_p is the volume fraction of the phase of interest. For the ionomer or membrane phase,

$$\varepsilon_M = (1 - \varepsilon_s) f_M \tag{4}$$

where f_M is the volume fraction of ionomer in the pore space and ε_s is the volume fraction of the solid volume of the porous electrode.

The diffusion coefficients in the gaseous phase are:

$$D_i^{eff} = \varepsilon_G^{1.5} \frac{1 - \omega_i}{\sum_{k \neq i} \frac{x_k}{D_{ik}}} \tag{5}$$

where x_k is the mole fraction of species *i.* The gas phase volume fraction, ε_G, is:

$$\varepsilon_G = (1 - \varepsilon_s)(1 - f_M)(1 - S) \tag{6}$$

where *S* is the CL or PTL liquid saturation.

Lastly, because water activity or chemical potential in the ionomer cannot be readily measured or observed, the simulated water activities are converted to membrane water content, λ, by the following semi-empirical expression:[2]

$$\lambda = (1 - S_M)\lambda_V + \lambda_L S_M \tag{7}$$

$$\lambda_V = 30.752a_v^3 - 41.194a_v^2 + 21.141a_v \tag{8}$$

where λ_V is the water content of vapor equilibrated AEM and λ_L is the water content of a liquid equilibrated AEM (set to a constant value of 17)[3].

Source Term Definitions

$R_{CT,i}$, represents the molar source terms of species *i* due to charge transfer reactions, respectively,

$$R_{CT,i} = -\sum_k \frac{s_{i,k} i_k}{n_k F} \tag{9}$$

where $s_{i,k}$ is the stoichiometric coefficient of species *i* in reaction *k*, and n_k is the number of electrons transferred in reaction *k*.

For water vapor, an additional phase-transfer term related to the modeled transfer of water from the liquid or ionomer to the gas phase is required,

$$R_{PT,w,G} = -A_s k_{MT,v}\left(\frac{RH}{100} - a_w\right) - k_{MT}^{'}(RH - 100\%)[H_0\left(\frac{p_L}{p_{ref}}\right) + H_0(RH - 100\%)] , \tag{10}$$

where $k_{MT,v}$ is the mass-transfer coefficient between the vapor phase and hydrated ionomer phase, *RH* is the relative humidity, and p_L is the bulk pressure of the liquid phase. The first term in the above equation describes mass transfer between vapor phase and the hydrated CL ionomer. The second term describes water evaporation or condensation in both the CL and PTL. A mass transfer coefficient of $k_{MT}^{'} = 10^7 mol\, m^{-3} s^{-1}$ and implementation of the Heaviside step function $H_0(x)$ ensure that RH is always 100% when liquid water is present and that the RH never exceeds 100%. Similarly, for liquid phase water:

$$R_{PT,w,L} = -A_s k_{MT,L}(p_L - p_{L,M}) + k_{MT}^{'}(RH - 100\%)[H_0\left(\frac{p_L}{p_{ref}}\right) + H_0(RH - 100\%)] , \tag{11}$$

where $p_{L.M}$ is the pressure of liquid water in the membrane. Again, the first term describes transfer between the liquid and ionomer phases, and the second term describes evaporation or condensation.

Additionally, the phase-transfer source term associated with water in the ionomer phase is given as

$$R_{PT,w,M} = A_s k_{MT,L}(p_L - p_{L,M}) + A_s k_{MT,v}\left(\frac{RH}{100} - a_w\right), \quad (12)$$

It is important to note that, while for vapor- or liquid-phase water there was no charge-transfer source term, there is a source term associated with the consumption of water by charge-transfer reactions in the ionomer phases. The phase-transfer source term associated with water in the ionomer phase is given as

$$R_{PT,w,M} = A_s k_{MT,L}(p_L - p_{L,M}) + A_s k_{MT,v}\left(\frac{RH}{100} - a_w\right), \quad (13)$$

Q_p describes the source term into or out of a given phase *p*. For the gas phase, the expression is

$$Q_G = M_W R_{PT,w,G} + \sum_{i \neq CO_2, H_2O, N_2} M_i R_{CT,i} \quad (14)$$

For liquid phase,

$$Q_L = M_W R_{PT,w,L} \quad (15)$$

Table S1: Parameter values for simulation.

Parameter	Value	Unit	Ref
**Geometry**			
H	360	μm	
H_{Chan}	109	μm	
L_{CL}	5	μm	
L_{PTL}	100	μm	
L_{Mem}	100	μm	
L_{Chan}	50	μm	
**Charge Transport**			
κ_L	20.6	mS cm^{-1}	[4]
σ_s	$\{220\ (PTLs)\ 100\ (CLs)$	S m^{-1}	[4]
ε_s	$\{0.47\ (PTLs)\ 0.5\ (CLs)$		[4]
f_M	0.4		[4]
**Reaction Kinetics**			
A_s	$1\ x\ 10^6$	m^{-1}	[4]
$i_{o,CO}$	$3.48\ x\ 10^{-14}$	mA cm^{-2}	[1]

$\alpha_{c,CO}$	1		1
$\gamma_{CO_2,CO}$	1.5		5
$U_{0,CO}$	-0.11	V vs. RHE	6
i_{o,H_2}	$5.09\ x\ 10^{-10}$	mA cm^{-2}	1
α_{c,H_2}	0.44		1
γ_{CO_2,H_2}	0		5
U_{0,H_2}	0	V vs. RHE	6
$i_{o,O_2,base}$	$4.78\ x\ 10^{-8}$	mA cm^{-2}	1
$i_{o,O_2,acid}$	$1.11\ x\ 10^{-8} exp(-0.4pH_0)$	mA cm^{-2}	1
α_{a,O_2}	1.5		1
U_{0,O_2}	1.23	V vs. RHE	6
**Species Transport**			
H_{CO_2}	34	mM atm^{-1}	7
ξ_A^M	-1		4

Tortuosity Calculations

To calculate the tortuosity of the ionically conducting medium, we consider an applied potential for which the membrane is fully hydrated, and there are no variations in conductivity across the domain, $V_{app} = 1.6\,V$. The ionomer conductivity at this potential is 20.6 $mS\ cm^{-1}$. The power loss due to ohmic losses throughout the modified ionomer domain is calculated as:[8]

$$P_{ohmic}^{real} = \int\int_{AEM} \frac{i_l \cdot i_l}{\kappa_{AEM}} dA \tag{16}$$

where i_l is the local ionic current density vector, and $\kappa_{AEM}$is the local AEM conductivity. The calculated power loss represents the loss of power through the ionomer, accounting for the tortuous pathway of the ions around the water channel.

If there were no tortuous pathway, the power loss would be the ideal power loss:

$$P_{ohmic}^{ideal} = \frac{i_l^2 A}{\kappa_{AEM}} \tag{17}$$

where A is the through-plane area of the AEM. We can use the above expression to determine an effective conductivity of the ionomer using the real ohmic power loss. Essentially, this value indicates the corresponding conductivity of a membrane without a channel that has the same ohmic power loss:

$$\kappa_{AEM}^{eff} = \frac{i_l^2 A}{P_{ohmic}^{real}} \tag{18}$$

This value is reduced compared to the bulk AEM conductivity of 20.6 $mS\ cm^{-1}$..

To obtain the tortuosity (τ), we divide the bulk AEM conductivity of 20.6 $mS\ cm^{-1}$ by the calculated effective conductivity. This value provides an average increase in the path length for each of the AEMs with internal channels.

$$\tau = \frac{20.6\ mS\ cm^{-1}}{\kappa_{AEM}^{eff}} \tag{19}$$

The calculated effective conductivities and tortuosities for the bilayer AEM with varying channel spacings (*H*) can be found in Table S2 below.

Table S2: Table of effective conductivity and tortuosity as a function of table spacing as calculated by equations 16-19.

H **(Channel Spacing,** μ**m)**	κ_{AEM}^{eff} **(Effective Conductivity, mS cm^{-1})**	τ **(Tortuosity)**
360	8.6	2.39
180	13.1	1.57
90	15.8	1.31

Supplementary Experimental Methods

Testing the Ionomer

To test the imidazolium-functionalized poly(phenylene oxide) polymer's performance (conductivity and electrochemical response) under electrolysis conditions, an electrolyzer with 5 cm^2 area from Dioxide materials, which has a serpentine flow channel on both the anode and cathode endplates, was utilized. The cathode was a 6.25 cm^2 gas-diffusion electrode (GDE) sputtered with 100 nm-thick silver. A 100 nm thick Ag catalyst layer was sputtered on top of the microporous layer of a Sigracet 38 BC by direct-current magnetron sputtering. The anode was a Ni foam (3x3 cm). The cell voltage was stable at 3.6 V and the average faradaic efficiency was 74%.

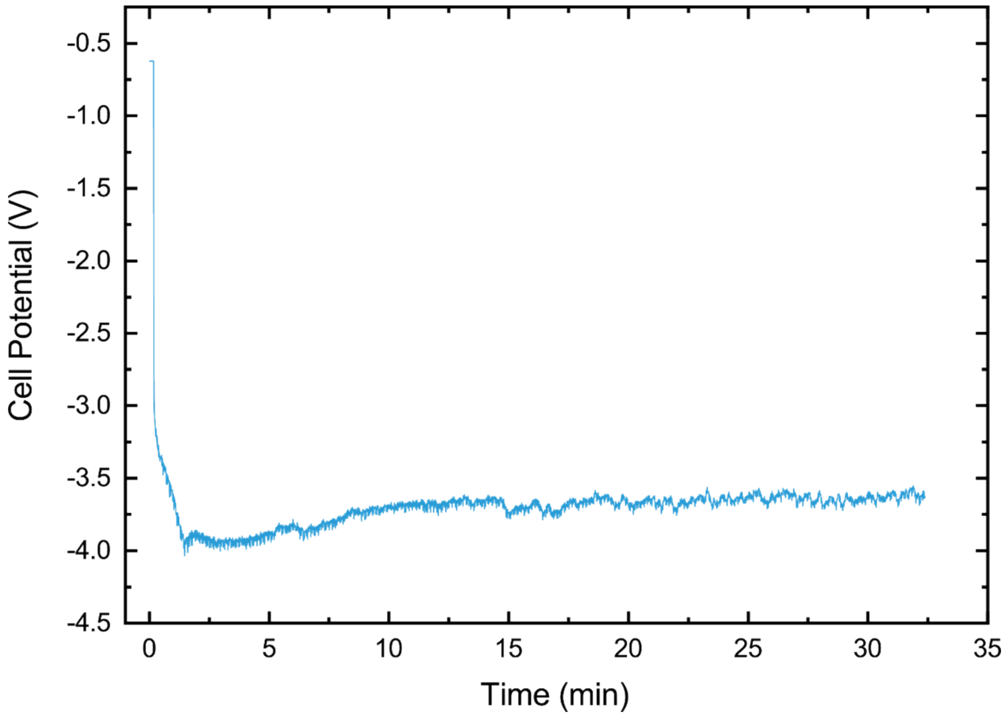

Figure S2 - Electrolysis at 100 mA/cm², in zero-gap configuration using a 32 µm thick PPO membrane

Custom-made Electrolyzer

Our custom-made electrolyzer chassis was assembled from two milled polyetheretherketone (PEEK) plates. One of the plates had four entrances: an inlet and outlet for the flow field, and two connections for electrodes. Gold spring contact electrodes were used to apply voltage/current to the electrolyzer. The other plate (Figure S1) had six entrances: the same four as the first plate, and an extra inlet and outlet for the membrane internal microchannels. The cathode was a silver-coated GDE and the anode was an iridium-oxide coated GDE. Both of these GDEs were 2.25 cm^2 and were prepared by sputter coating (AJA International Sputter Machine) pure Ag and Ir, respectively, onto a Toray TGP-H-060 porous carbon paper (Alfa Aesar).

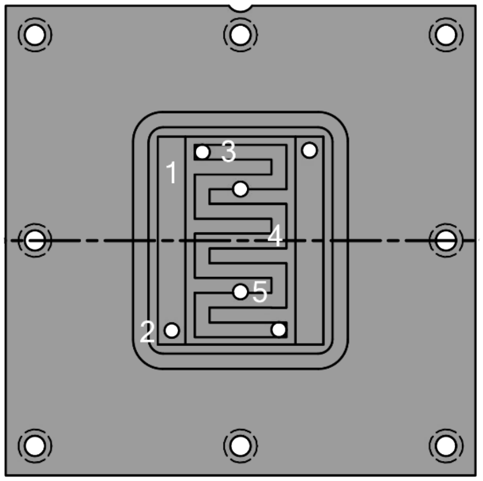

Figure S3 – Schematic of one plate of the custom-made electrolyzer schematic. 1 - seat for gasket that ensures the fluid in the internal channels does not crossover to the flow-field; 2- inlet/outlet for internal membrane microchannels; 3 - inlet/outlet for plate flow-field; 4 – flow-field; 5 – entrance for electrode connection. The other plate is analogous, but does not have the inlet/outlet for membrane microchannels (2).

Resistance Measurements

To measure the membrane resistance with different electrolyte concentrations in the microchannels, two T-junctions were added in the inlet tubing close to the electrolyzer plates. In those junctions, two leakless Ag/AgCl reference electrodes were connected. This way, the voltage drop was measured between the electrolyte inlets. Since current flows only between the electrodes, and the membrane separates the electrodes, the voltage drop across the membrane is measured. The operating conditions can be found in the main text.

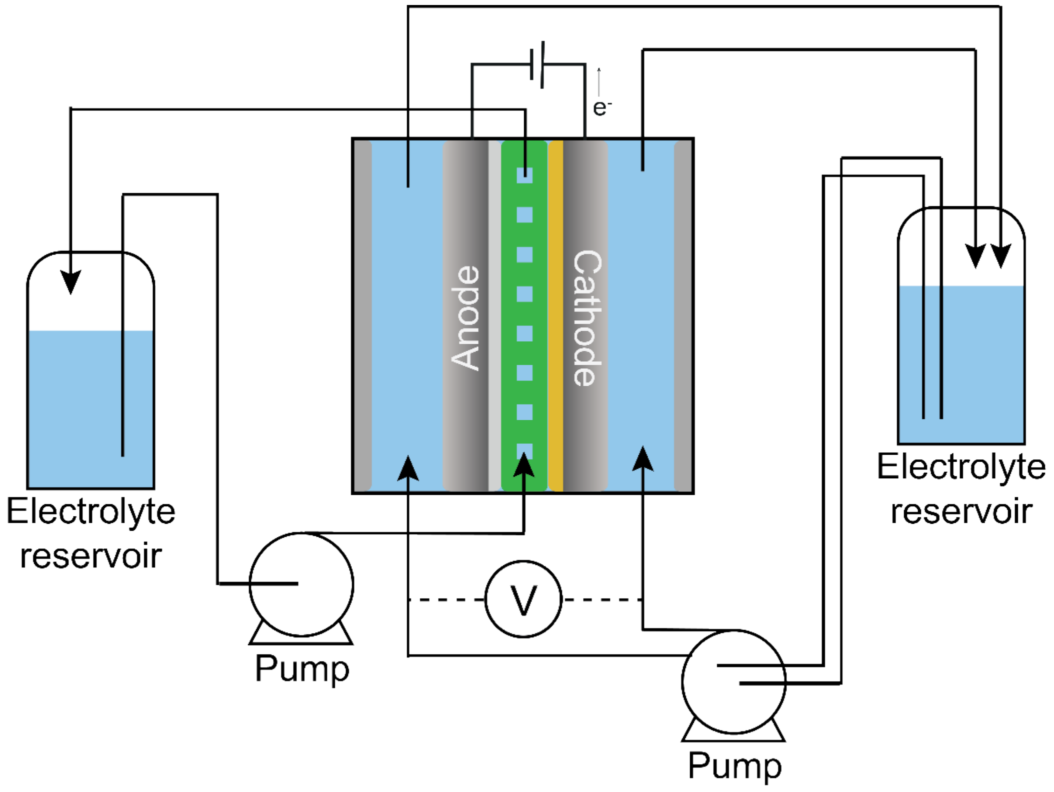

Figure S4 - Schematic of the membrane resistance measurement. The AEM with internal microchannels (green) is between the anode and the cathode. $KHCO_3$ solutions of varying concentrations were introduced into the left electrolyte reservoir, and a 0.1 M $KHCO_3$ solution was introduced into the right reservoir.

Electrolysis Experiments

A humidified CO_2 stream was introduced into the cathode side of the reactor for the electrolysis experiments. The gas stream was humidified by bubbling dry CO_2 through a sparger

into a DI water column at room temperature, and the relative humidity was measured with a humidity sensor. A 0.1M KOH solution or a humidified N_2 stream were fed as reactants for the oxygen evolution reaction on the anode side. Linear sweeps of the voltage from 0 to −4 V, while measuring the current, were made in triplicate to assess the effect of the different electrolyte concentrations in the microchannels.

To quantify the K^+ crossover to the cathode side, 0.1 M KOH was fed into the anode channel, and a constant current of 5 mA/cm^2 was applied for 73 minutes. Aliquots of the anolyte and of the electrolyte in the internal membrane channels were taken before and after current was applied. At the end of the experiment, the serpentine flow channels in the cathode plate were rinsed (collecting the liquid), and the cathode GDE was placed in 30 mL of aqueous solution containing 5 mL isopropanol and approximately 1.5 mL of concentrated HCl. This solution was chosen to counter the GDE's hydrophobicity and to dissolve any potassium salts that had deposited. Five aliquots were analyzed using ion chromatography and a mass balance on the K^+ was made.

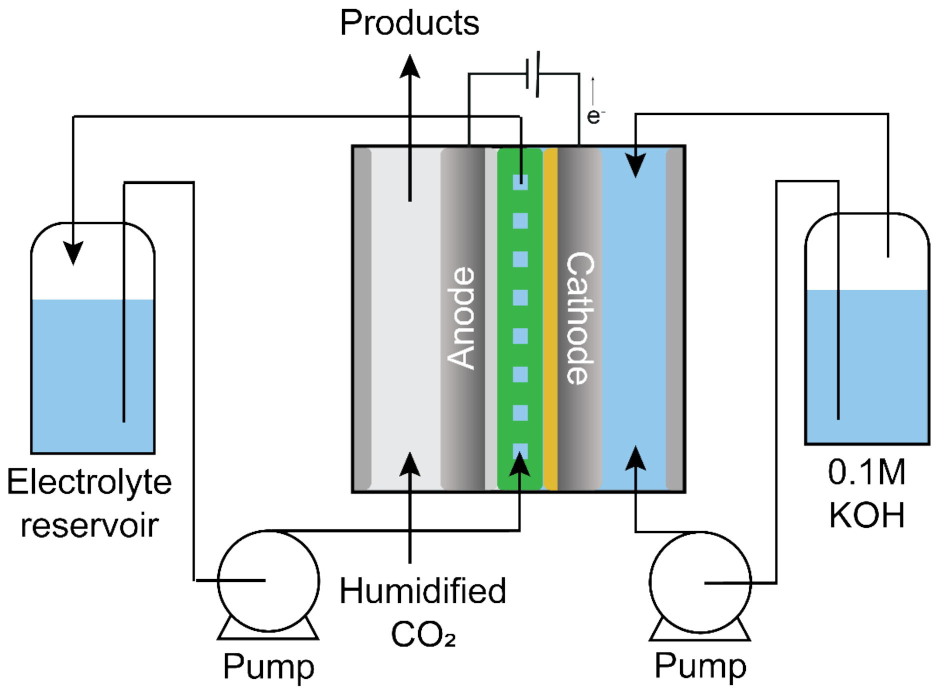

Figure S5 - Electrolysis setup.

Supplementary Results

Water Content Distribution at 2.5 V for Base Case (Spacing of 360 µm)

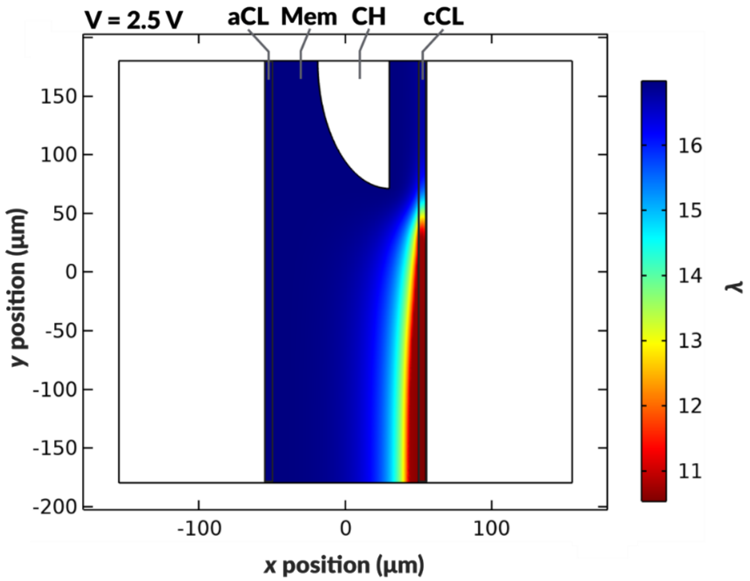

Figure S6 – Water content distribution in the bilayer AEM with a curved channel geometry and spacing of H = 360 µm. The applied potential for this simulation is 2.5 V.

Water Content Distribution for AEM with No Microchannels at Various Applied Voltages

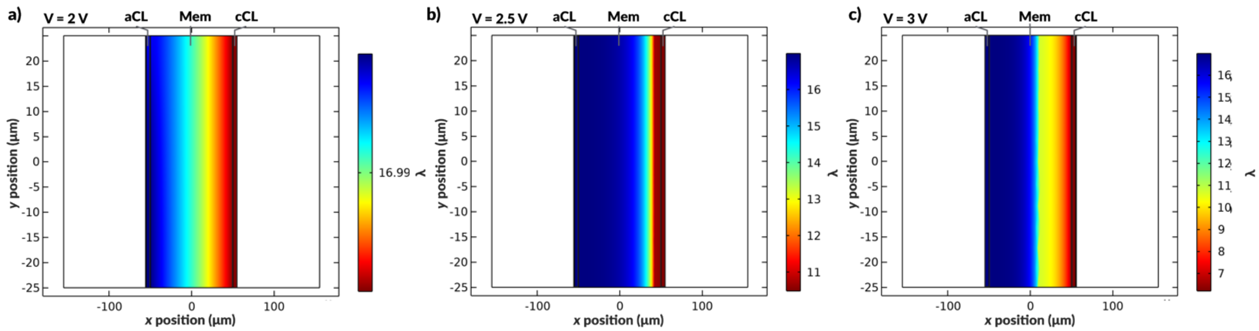

Figure S7 – Water content distribution in the AEM with no internal microchannels for ***(a)*** *2 V,* ***(b)*** *2.5 V* ***(c)*** *3 V applied potentials.*

Water Content Distribution for bilayer AEM with *H* = 180 μm at Various Applied Voltages

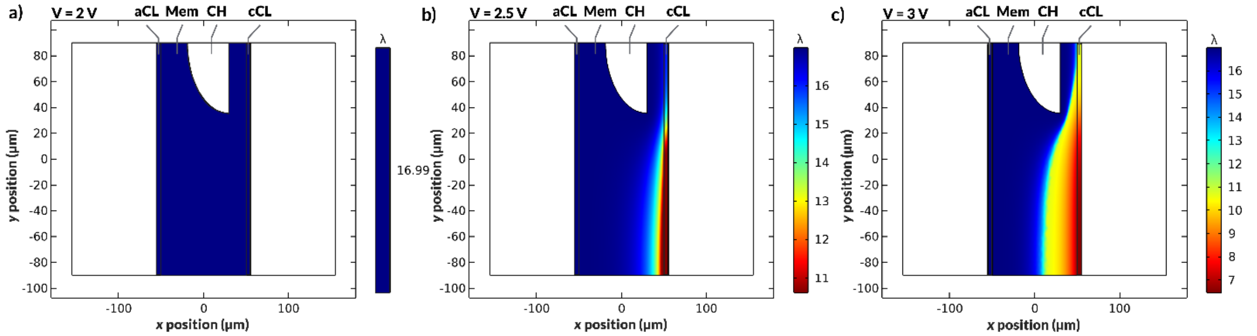

Figure S8 – Water content distribution in the bilayer AEM with a curved internal channel with channel spacing of H = 180 μm *for* ***(a)*** *2 V,* ***(b)*** *2.5 V* ***(c)*** *3 V applied potentials.*

Water Content Distribution for bilayer AEM with *H* = 90 μm at Various Applied Voltages

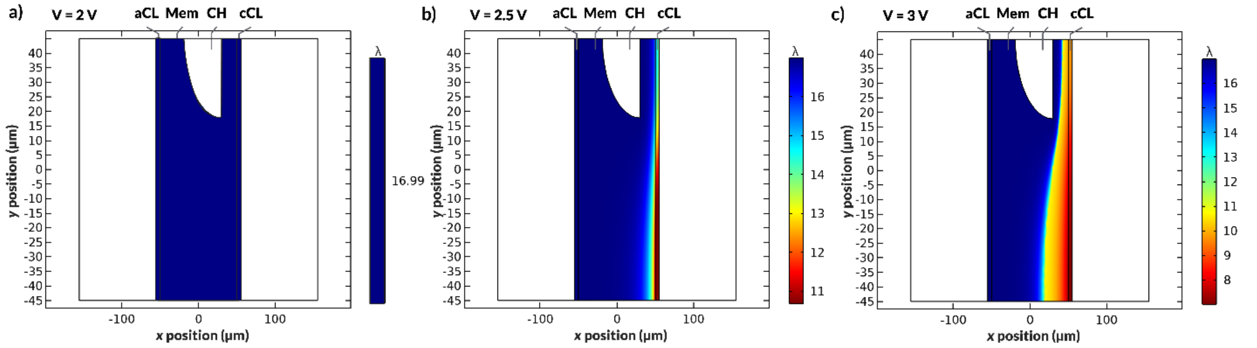

Figure S9 – Water content distribution in the bilayer AEM with a curved internal channel with channel spacing of H = 90 μm *for* ***(a)*** *2 V,* ***(b)*** *2.5 V* ***(c)*** *3 V applied potentials.*

Local Water Content and Current Density for AEMs Where the Microchannel Contacts the CL

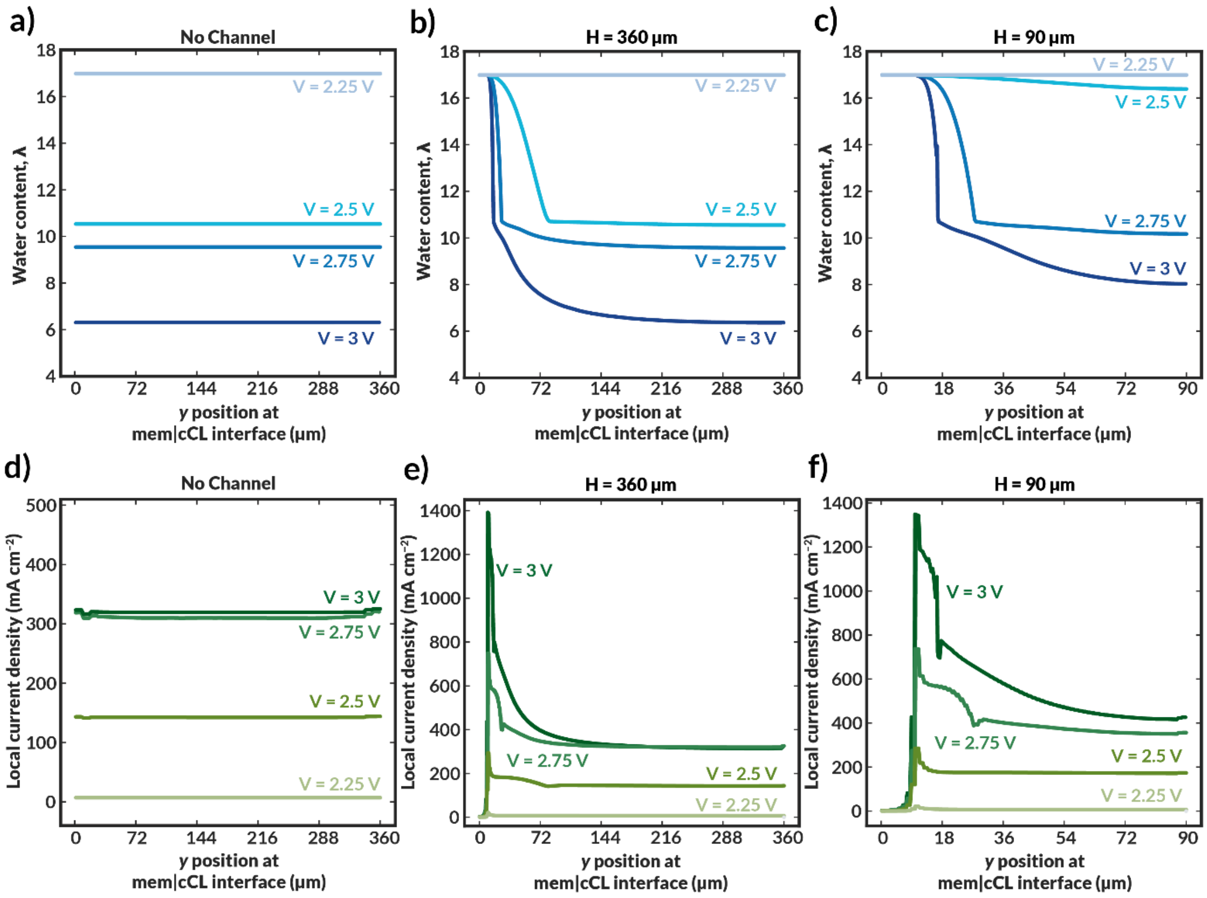

*Figure S10 – **(a-c)** Local water content of the ionomer within the cathodic CL averaged across the CL thickness for varying channel geometries and spacings for AEMs where the microchannel is in directly contact with the CL. **(d-f)** Local current density within the cathodic CL averaged across thickness of the CL for varying channel geometries and spacings for AEMs where the microchannel is in direct contact with the CL.*

Electrolysis Experiments

Table S3: Average faradaic efficiency observed during electrolysis with different electrolyte concentrations in the internal membrane channels, with a liquid anolyte and at 5 mA/cm^2.

C_{in} (mM)	**FE (Faradaic Efficiency, %)**
No Channels	58
DI Water	57
10	55
100	62
Average	58

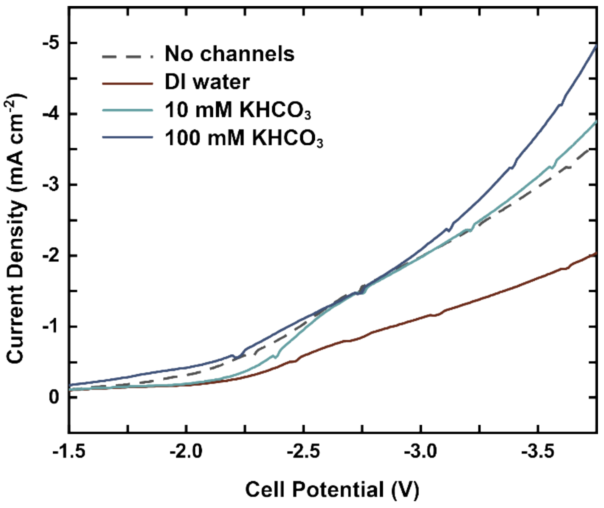

Figure S11 - Galvanostatic linear sweep experiments with different electrolyte concentrations inside the membrane channels in full MEA configuration, with hydrated N_2 gas as anolyte

Donnan model for K^+ cross-over

We can consider an ion exchange membrane, with fixed charge density X, immersed in an aqueous solution with a salt concentration of c. Based on the classical Donnan model, the concentration of co-ions and counter-ions in the membrane, $c_{counter}$ and c_{co} satisfy the following two equations ([7]):

$$c_{counter} \cdot c_{co} = c^2 \quad (20)$$

$$c_{counter} + c_{co} = \sqrt{X^2 + (2c)^2} \tag{21}$$

That means the ratio between co-ions and counter-ions can be rewritten as:

$$\frac{c_{co}}{c_{counter}} = \frac{c^2}{X\sqrt{X^2 + (2c)^2} - c^2} \tag{22}$$

That means the ratio $\frac{c_{co}}{c_{counter}}$ increases as c increases, which is equivalent to a relatively high sorption coefficient of co-ions in the membrane at high external concentration. This has also been confirmed by experiments.[9]

Supplemental References

1 L. C. Weng, A. T. Bell and A. Z. Weber, *Energy Environ. Sci.*, 2019, **12**, 1950–1968.

2 A. Z. Weber and J. Newman, *Modeling transport in polymer-electrolyte fuel cells*, 2004, vol. 104.

3 J. Peng, A. L. Roy, S. G. Greenbaum and T. A. Zawodzinski, *J. Power Sources*, 2018, **380**, 64–75.

4 L. C. Weng, A. T. Bell and A. Z. Weber, *Energy Environ. Sci.*, 2020, **13**, 3592–3606.

5 M. R. Singh, J. D. Goodpaster, A. Z. Weber, M. Head-Gordon and A. T. Bell, *Proc. Natl. Acad. Sci. U. S. A.*, 2017, **114**, E8812–E8821.

6 J. C. Bui, E. W. Lees, L. M. Pant, I. V. Zenyuk, A. T. Bell and A. Z. Weber, *Chem. Rev.*, , DOI:10.1021/acs.chemrev.1c00901.

7 C. Press, *CRC handbook of chemistry and physics*, Cleveland, Ohio, 1977.

8 M. R. Gerhardt, L. M. Pant, J. C. Bui, A. R. Crothers, V. M. Ehlinger, J. C. Fornaciari, J. Liu and A. Z. Weber, *J. Electrochem. Soc.*, 2021, **168**, 074503.

9 A. H. Galama, J. W. Post, M. A. Cohen Stuart and P. M. Biesheuvel, *J. Memb. Sci.*, 2013, **442**, 131–139.
